# Ischemia-Reperfusion Increases TRPM7 Expression in Mouse Retinas

**DOI:** 10.3390/ijms242216068

**Published:** 2023-11-08

**Authors:** Natalia Martínez-Gil, Oksana Kutsyr, Laura Fernández-Sánchez, Xavier Sánchez-Sáez, Henar Albertos-Arranz, Carla Sánchez-Castillo, Lorena Vidal-Gil, Nicolás Cuenca, Pedro Lax, Victoria Maneu

**Affiliations:** 1 Departamento de Fisiología, Genética y Microbiología, Universidad de Alicante, 03690 San Vicente del Raspeig, Alicante, Spain; natalia.martinez.gil@ua.es (N.M.-G.); xsanchez@ua.es (X.S.-S.); henar.albertos@ua.es (H.A.-A.); carla.sanchez@ua.es (C.S.-C.); l.vidal@ua.es (L.V.-G.); cuenca@ua.es (N.C.); pedro.lax@ua.es (P.L.); 2 Departamento de Óptica, Farmacología y Anatomía, Universidad de Alicante, 03690 San Vicente del Raspeig, Alicante, Spain; oksana.kutsyr@ua.es (O.K.); laura.fs@ua.es (L.F.-S.)

**Keywords:** TRPM7, TRP, ischemia, reperfusion, retina degeneration

## Abstract

Ischemia is the main cause of cell death in retinal diseases such as vascular occlusions, diabetic retinopathy, glaucoma, or retinopathy of prematurity. Although excitotoxicity is considered the primary mechanism of cell death during an ischemic event, antagonists of glutamatergic receptors have been unsuccessful in clinical trials with patients suffering ischemia or stroke. Our main purpose was to analyze if the transient receptor potential channel 7 (TRPM7) could contribute to retinal dysfunction in retinal pathologies associated with ischemia. By using an experimental model of acute retinal ischemia, we analyzed the changes in retinal function by electroretinography and the changes in retinal morphology by optical coherence tomography (OCT) and OCT-angiography (OCTA). Immunohistochemistry was performed to assess the pattern of TRPM7 and its expression level in the retina. Our results show that ischemia elicited a decrease in retinal responsiveness to light stimuli along with reactive gliosis and a significant increase in the expression of TRPM7 in Müller cells. TRPM7 could emerge as a new drug target to be explored in retinal pathologies associated with ischemia.

## 1. Introduction

The retina is a tissue with a high oxygen requirement, making it highly sensitive to blood supply. Ischemia is present in some of the most prevalent diseases, causing visual impairment and blindness [1,2]. Among them we find (i) retinal vascular occlusions, caused either by thrombosis or embolization, which are the second leading cause of retinal vascular disorders [3]; (ii) retinopathy of prematurity, a vasoproliferative disorder that is the leading cause of visual impairment and blindness worldwide during childhood and whose vascular changes cause retinal ischemia [4]; (iii) diabetic retinopathy, a common complication of diabetes mellitus where different levels of retinal ischemia exist [1,2,5]; and (iv) neovascular glaucoma, most frequently caused by severe retinal ischemia [6].

During an ischemic event, the pathological increase in neurotransmitters such as glutamate becomes toxic to cells, triggering excitotoxicity, which is considered the primary mechanism of neuronal dysfunction and cell death. Accordingly, the use of N-methyl-D-aspartate (NMDA) receptor blockers to prevent cell damage demonstrates neuroprotection on its own. However, the fact that treatments with antagonists of glutamatergic receptors were unsuccessful in clinical trials in stroke or ischemia reveals the need to explore additional signaling pathways involved in neuronal damage to find new therapeutic agents [7,8,9,10]. At present, neuronal death induced by ischemia is accepted to be a result of a combination of glutamate-mediated excitotoxicity together with non-glutamate cell death pathways, mainly mediated by an increase in intracellular calcium. Several members of the transient receptor potential channels (TRPs), such as non-glutamatergic contributors involved in cellular death after ischemic injury, are arising as alternative sources responsible for the calcium overload [9,11,12,13,14,15].

TRPs are a superfamily of ion channels first identified in the photoreceptor cells of Drosophila [16]. In humans, they comprise six families that include nearly 30 different known members. TRPs act as transductors and transmitters of visual information, operating as primary detectors of chemical and physical stimuli, and as secondary effectors of metabotropic and ionotropic receptors. They are highly susceptible to non-visual stimuli and mediate the cellular response to changes in mechanical pressure, temperature, pH, osmolarity, inflammation and oxidation, among others. Due to their function as calcium channels, they control the membrane potential in resting excitable and non-excitable cells and tune light-dependent and -independent functions of retinal circuits [15]. Mammalian retinas express most of the TRP isoforms and each retinal cell type expresses one or more different subtypes [15,17]. To date, several studies have shown the involvement of some members of the TRP family in ON-bipolar signaling, intrinsically photosensitive ganglion cell phototransduction and the osmoregulation, mechanotransduction, and regulation of the inner and outer blood–retinal barriers [15].

TRPM7 has been related to the damage induced by ischemia in different tissues such as brain, kidney and heart tissues [1,14,18,19,20,21]. In the brain, TRPM7 is a critical mediator of anoxic neuronal death in ischemic and hypoxic injury. In animal models, ischemia-induced oxidative stress leads to an increase in TRPM7 expression in the brains of mice and ionic currents are upregulated, probably contributing to neurotoxicity, whereas the inhibition or suppression of TRPM7 exerts neuroprotection [18]. In the kidneys, TRPM7 mRNA and protein levels are increased after just one hour of renal ischemia in rats [19,20]. TRPM7 was also upregulated in a rat model of myocardial ischemia and reperfusion [22].

Our main objective in this work was to evaluate the possible implication of TRPM7 in retinal dysfunction and cell death occurring after ischemia. Changes in the retinal function as well as in the tissue expression level and pattern of TRPM7 expression were assessed in a murine model of ischemia-reperfusion (I/R). In our study, the significant increase in the expression of the TRPM7 channel in ischemic retinas suggests that TRPM7 should be considered as a potential therapeutic target in ischemic retinal pathologies, as diabetic retinopathy or glaucoma.

## 2. Results

### 2.1. Effects of Ischemia-Reperfusion on Retinal Function

**Figure 1:**
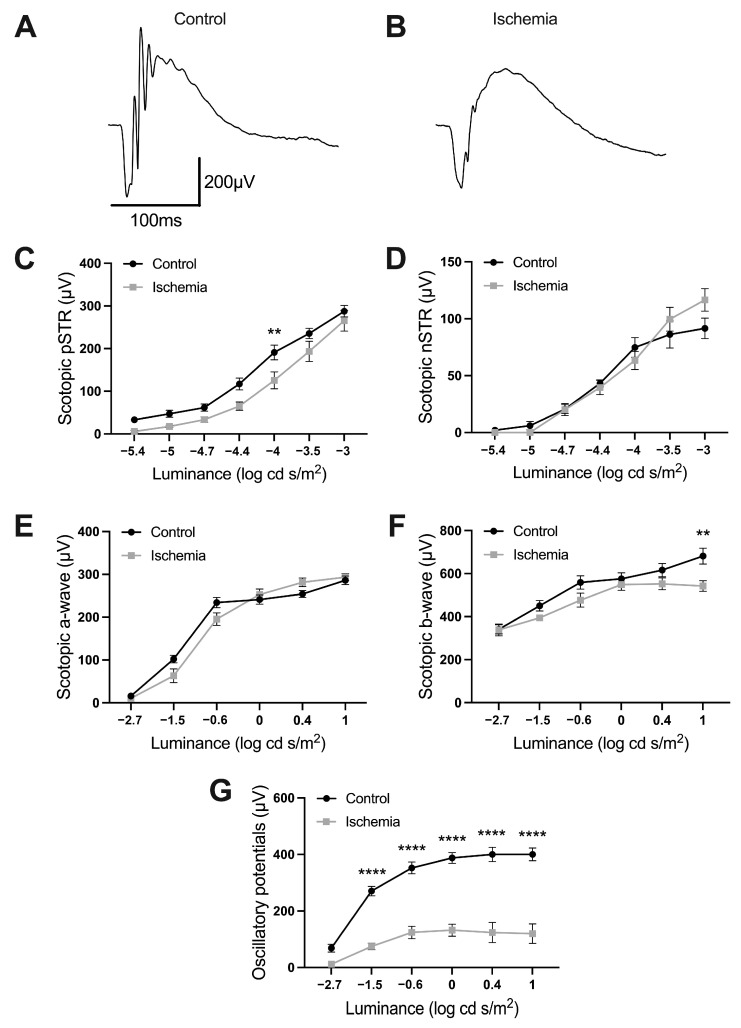
Effect of ischemia/reperfusion on retinal function from control non-ischemic (**A**,**C**–**G**) and ischemic C57BL/6J mice (**B**,**C**–**F**) after a 30 min period of ischemia and 72 h of reperfusion (*n* = 5). (**A**,**B**) Representative dark-adapted electroretinogram (ERG) intensity responses to 1 log cd·s/m^2^ flashes. (**C**–**F**) Luminance–response curves: positive scotopic threshold response (pSTR) (**C**), negative scotopic threshold response (nSTR) (**D**), a-wave (**E**), b-wave (**D**), and oscillatory potentials (**G**). Two-way ANOVA and Bonferroni post hoc test. ** *p* < 0.01, **** *p* < 0.0001.

To assess the impact of a mild period of ischemia (30 min) on the functional activity of the retina, electroretinographic responses induced by a brief flash under scotopic conditions were recorded in ischemic eyes as well as in control animals (without the ischemia process) 72 h after the induction of ischemia. As shown in Figure 1, ischemia affected the b-wave and the positive scotopic threshold response (pSTR) responses to light flashes. More precisely, the b-wave amplitude at higher stimulus intensities (1 log·cd·s/m^2^) in ischemic mice was 79.6% of the value observed in control animals (681.4 to 542.4 µV in control and ischemic eyes, respectively) (ANOVA, Bonferroni’s test, *p* < 0.01) (Figure 1A,B,F). pSTR values at −4 log·cd·s/m^2^ in ischemic mice were 65.8% of the value observed in control animals (191.0 vs. 125.6 µV in control and ischemic eyes, respectively) (ANOVA, Bonferroni’s test, *p* < 0.001) (Figure 1A–C). Moreover, the oscillatory potentials were highly affected in ischemic eyes. The amplitude of oscillatory potentials at higher stimulus intensities (1 log·cd·s/m^2^) in ischemic mice was 29.9% of the value observed in control mice (400.2 vs. 119.6 µV in control and ischemic eyes, respectively) (ANOVA, Bonferroni’s test, *p* < 0.0001) (Figure 1A,B,G). The amplitudes of the a-wave and negative scotopic threshold response (nSTR) were not significantly affected in these conditions (Figure 1D,E).

### 2.2. Effects of Ischemia-Reperfusion on Retinal Integrity

**Figure 2:**
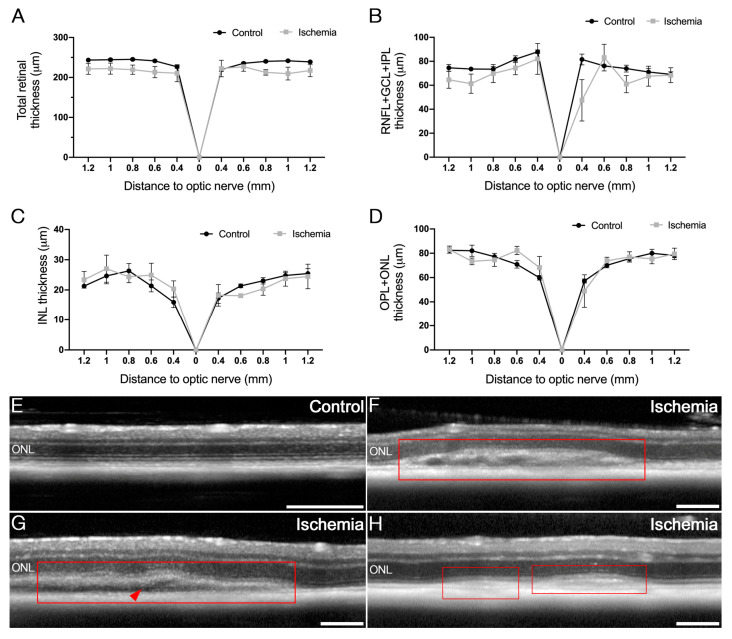
Effect of ischemia/reperfusion on retinal structure. (**A**–**D**) Thickness of the retinal layers analyzed by OCT: thickness of the total retina (**A**), the RNFL-GCL-IPL complex (**B**), the INL (**C**) and the OPL+ONL (**D**) measured at different locations from the optic nerve (0 on the graphs) in control (non-ischemic) (*n* = 5) and I/R mice (*n* = 5). (**E**–**H**) OCT images from control non-ischemic C57BL/6J (**E**) and different ischemic mice (**F**–**H**) after a 30 min period of ischemia and 72 h of reperfusion. Hyperreflective areas at the level of the outer retinal layers (**F**–**H**, red squares) and mild retinal detachments (**G**, red arrow) existed in the ischemic mice. Scale bars: 200 µm. RNFL: retinal nerve fiber layer; GCL: ganglion cell layer; IPL: inner plexiform layer; INL: inner nuclear layer; OPL: outer plexiform layer; ONL: outer nuclear layer.

In order to assess the structural alterations of the retina, optical coherence tomography (OCT) images were compared before and 72 h after ischemia. The thicknesses of the different retinal layers in the optic nerve were similar between the non-ischemic control and ischemic groups (Figure 2A–D), although a slight reduction in the total retinal thickness was observed (Figure 2A). Moreover, morphological changes were specifically found in the ischemic mice; hyperreflective areas of variable size localized in the outer retinal bands (including the external section of photoreceptors cells and the retinal pigment epithelium) existed in all ischemic animals (Figure 2F–H, red squares). Some of these areas also presented a slight retinal detachment (Figure 2G, arrow). These changes were not observed in control animals, which were cannulated but in which no ischemia was performed.

**Figure 3:**
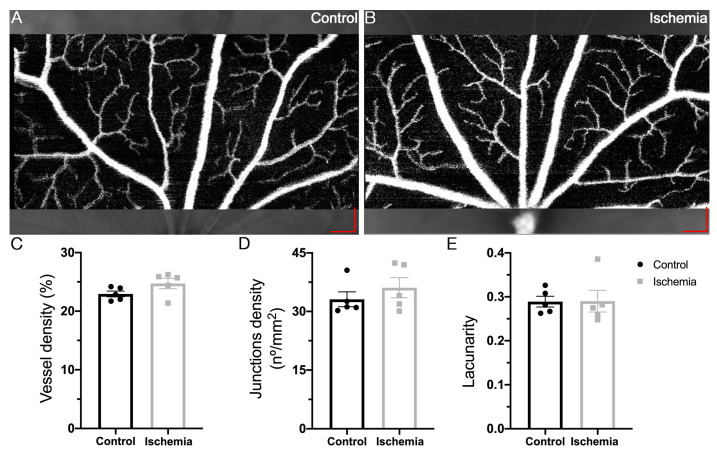
Effect of ischemia/reperfusion on retinal blood vessels. (**A**,**B**) OCTA images from the superficial vascular plexus of non-ischemic control (**A**) and ischemic mice (**B**) (scale bars, in red: 200 µm). (**C**–**E**) Vascular parameters analyzed with AngioTool^®^ software showed no differences among control (*n* = 5) and ischemic mice (*n* = 5).

The retinal vessels of the superficial vascular plexus from OCT-angiography (OCTA) images were analyzed with AngioTool^®^ software. The vascular density, the junction density and the lacunarity in the I/R mice showed no differences compared to the control group (Figure 3).

### 2.3. TRPM7 Is Expressed in Müller Cells of Mouse Retinas

**Figure 4:**
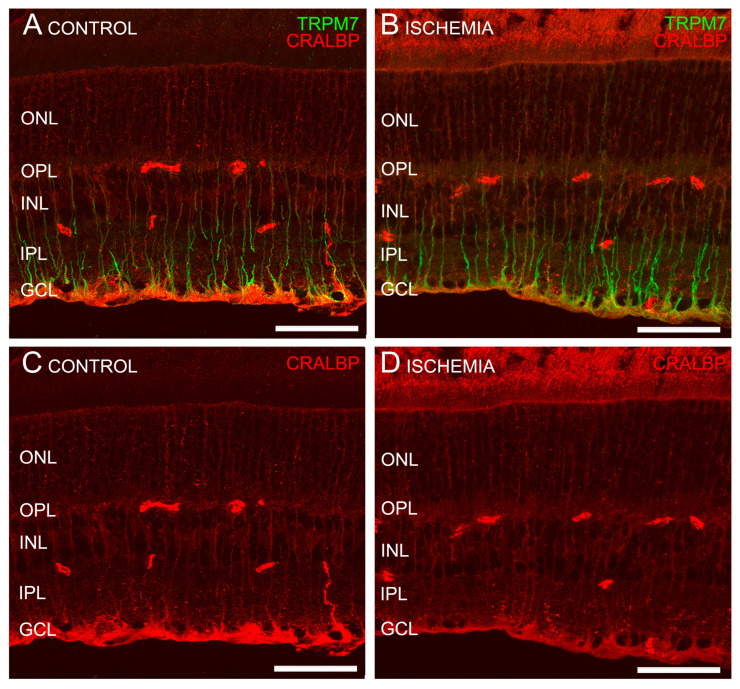
TRPM7 expression in Müller cells. Cross-sectional retinas from a representative control (non-ischemic) (**A**,**C**) and ischemic mouse (**B**,**D**) stained with anti-TRPM7 antibody (green) (**A**,**B**) and anti-CRALBP antibody (red) (**A**–**D**). In these images, TRPM7 is shown to colocalize with Müller cells, which are CRALBP-positive. Scale bar: 50 µm.

**Figure 5:**
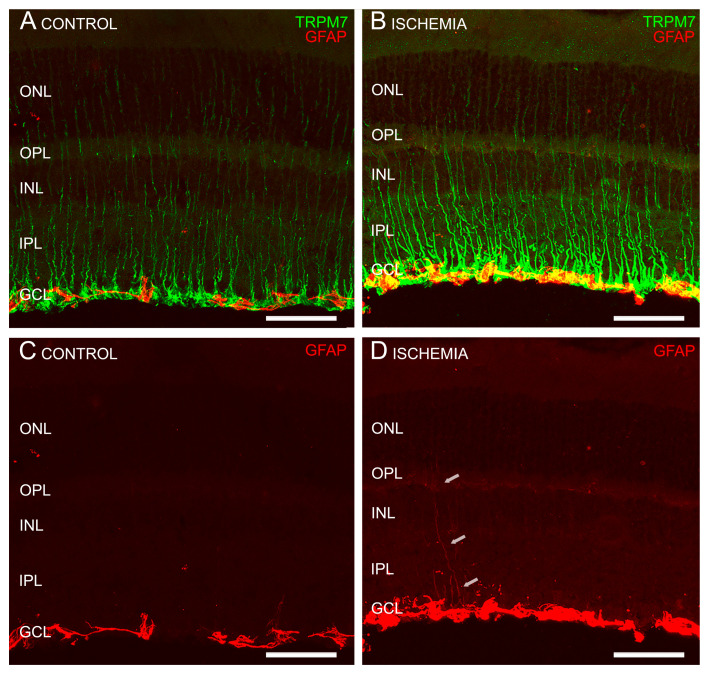
TRPM7 expression in mouse retinas. Cross-sectional retinas from a representative control mouse (non-ischemic) (**A**,**C**) and a ischemic one (**B**,**D**) stained with anti-TRPM7 antibody (green) (**A**,**B**) and anti-GFAP antibody (red) (**A**–**D**). TRPM7 is not expressed in the astrocytes in the control mouse retina (**A**,**C**), whereas in ischemic eyes (**B**,**D**), GFAP immunofluorescence is detected in the end-feet of Müller cells and also in the stalk due to gliosis (white arrows). Scale bar: 50 µm.

Double immunostaining with anti-TRPM7 and anti-CRALBP antibodies (which stain retinal pigment epithelial cells and Müller cells) showed a colocalization of TRPM7 immunoreactivity with Müller cells (CRALBP positive) in their end-feet (Figure 4). GFAP antibodies are used as a marker for astrocytes and reactive Müller cells. Double immunostaining with anti-TRPM7 and anti-GFAP antibodies discarded TRPM7 expression in astrocytes (GFAP positive) in control (non-gliotic) retinas (Figure 5A,C). In ischemic mice, an increased expression of GFAP in Müller cells was observed, indicating that gliosis of Müller cell was triggered by ischemia-reperfusion injury (Figure 5B,D). Under these conditions, TRPM7 was observed in the main process as well as in the stalk of the GFAP-immunopositive Müller cells (Figure 5B).

### 2.4. TRPM7 Expression Is Enhanced in Ischemic Retinas

**Figure 6:**
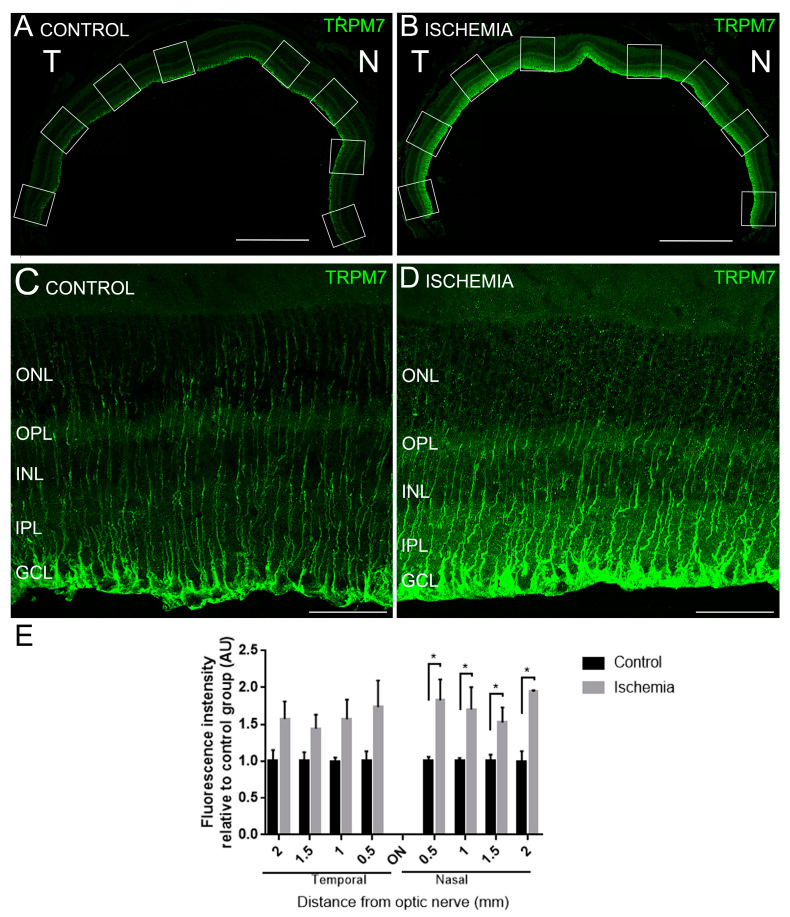
Effect of ischemia on the expression of TRPM7 in the retina. (**A**–**D**) Representative nasal-temporal cross-sections (**A**,**B**) and vertical cross-sections (**C**,**D**) of retinas from a control (non-ischemic) (**A**,**C**) and an ischemic mouse (**B**,**D**) stained with anti-TRPM7 antibody. Scale bars: 500 µm (**A**,**B**) and 50 µm (**C**,**D**). (**E**) Quantitation of the mean fluorescence intensity values throughout the nasal-temporal axis in both groups of mice. Mann–Whitney Test * *p* < 0.05. White boxes correspond to the regions where measurements were done.

TRPM7 channels were localized in Müller cells all throughout the nasal-temporal axis (Figure 6A,B). The quantification of the mean fluorescence intensity values in both groups of mice showed an increased expression of TRPM7 in ischemic eyes (Figure 6A–D). This difference reached significance in the nasal region of ischemic retinas (Figure 6E, *p* < 0.05).

## 3. Discussion

In this investigation, we have found that 30 min of retinal ischemia followed by 72 h of reperfusion upregulates TRPM7 channels. To the best of our knowledge, this is the first work that demonstrates the possible implication of TRPM7 channels in retinal degeneration after ischemic injury. These findings are in accordance with the results obtained after I/R procedures in kidneys [19,20,23], brains [14,18], and the myocardium [22].

Upregulation of TRPM7 channels after ischemia-reperfusion was accompanied by a reduction in the amplitude of oscillatory potentials in the ERGs, pointing to changes in the inner retina and slight decreases in the b-wave and nSTR amplitudes. The observed decrease in retinal light-evoked responses was less evident than that obtained in previous works, probably due to the shorter period of ischemia, as previous studies maintained ischemia for 45–90 min [24,25,26,27].

Even though no significant differences were observed in the retinal thickness by OCT, the ischemia-reperfusion process altered the outer retinal bands in all mice. These alterations in mice are scarcely described with imaging techniques and are considered retinal detachments [26]. Nevertheless, these patchy areas resemble what is histologically described as a hyperplasic or hypertrophic retinal pigment epithelium, along with a loss of the outer segments in other ischemic animal models [28,29]. Wilson et al. defined these lesions as result of the blood–retinal barrier breakdown associated with the ischemic process [30].

TRPM7 is a non-selective channel permeable to divalent cations that regulates the balance of intracellular Ca^2+^ and Mg^2+^ [31,32]. It mediates physiological functions such as cell growth, proliferation, migration and differentiation and it has been related to several pathological processes such as cancer, ischemic stroke or hypertension [33]. As a particular characteristic, only shared with its closely related family member TRPM6, TRPM7 contains both an ion channel and a serine-threonine kinase domain in its C-terminal portion, which have independent functions [31,34]. On the one hand, the movement of divalent cations across the ion channel can depolarize excitatory cells and cause intracellular calcium overload, with plenty of associated effects [35,36]. On the other hand, due to its kinase domain, TRPM7 can auto-phosphorylate or activate different substrates such as annexin-A1, myosin IIA or phospholipase Cγ2. In fact, TRPM7 has a regulatory role in MAPK signaling [37,38]. Other functions, as the regulation of volume-sensitive outwardly rectifying Cl^−^ channels (VSOR), require both the channel and the kinase activities of TRPM7 [39].

It is accepted that during an episode of ischemia, the levels of extracellular glutamate increase quickly in the affected area [40]. The massive release of glutamate leads to a calcium influx via N-methyl-D-aspartate (NMDA) receptors, and the intracellular calcium overload in turn activates calcium-dependent signaling cascades and increases the levels of oxygen and nitrogen species (nitric oxide, superoxide and peroxynitrite). These free radicals increase the TRPM7 conductance, resulting in a greater calcium influx and a higher production of oxygen and nitrogen free radicals, which imply more toxicity, eventually leading to cell death [11,18,41,42,43,44].

Considering our results, the expression of TRPM7 channels in healthy and ischemic retinas was located especially in Müller cells. Therefore, several mechanisms driving retinal cell death and survival should be considered. In physiological conditions, Müller cells support the synaptic activity of retinal neuronal cells by the reuptake and metabolism of neurotransmitters, contributing to the glutamate clearance. When a harmful stimulus occurs, reactive Müller cells exert a neuroprotective role by clearing the excess of extracellular glutamate, creating a scenario where a malfunction or downregulation of their transporters can contribute to neuronal degeneration [45]. We observed gliosis in ischemic eyes, supporting the idea of the contribution of Müller cells to the neurotoxic events triggered in the ischemic episode. We hypothesize that the increased expression of TRPM7 channels that is induced in I/R conditions in Müller cells probably exerts noxious effects in several ways. Some of them could involve the activation of calcium-dependent downstream cascades by the increased levels of intracellular calcium, and others may also be due to the increased phosphorylation of TRPM7 substrates. From our point of view, the increased number of TRPM7 channels in Müller cells could contribute to neuronal cell death in various ways, perhaps acting simultaneously, described in the following: (i) Inducing neurotoxic levels of extracellular glutamate. This can be achieved in two ways. On the one hand, as Müller cells in adult mice release glutamate by calcium-dependent mechanisms [45], increased intracellular calcium entry through TRPM7 channels could increase the extracellular levels of glutamate. On the other hand, as it has been described that Müller cell depolarization decreases the electrogenic glutamate uptake [46]; the increased cation influx into Müller cells can induce cellular depolarization and a decrease in glutamate uptake. (ii) Altering the release of neurotransmitters and neurotrophic factors by Müller cells. Glial cells release transmitters in a calcium- and SNARE (soluble N-ethylmaleimide-sensitive factor attachment protein receptor)-dependent manner. Experiments in mice show that impairing SNARE-dependent exocytosis in glial cells during ischemia increases neurotoxicity. Also, a decreased expression of functional SNARE in Müller cells protects the retinal function in the early phase of ischemia [25]. The impaired process of secretion in Müller cells could decrease the level of protective factors and increase neurotoxicity. In this sense, in retinal ischemia, retinal ganglion cells can resist a potential demise by secreting mediators that stimulate Müller cells to increase the production of neuroprotective factors, which counteract apoptotic cell death [47]. (iii) Increasing cell death signals in Müller cells. The excess calcium influx in cells can activate the apoptosis machinery of Müller cells [48]. Their death and the loss of their supportive functions would cause or aggravate a dysfunction and loss of neurons [46]. (iv) Increasing neuronal swelling. The ion fluxes in retinal neurons are associated with water movements that are mediated by aquaporin-4 water channels expressed by Müller cells [48]. In a model of rat cerebral ischemia, water permeability in astrocytes through aquaporin 4 was increased under high extracellular glutamate [49]. (v) Increasing calcium release from intracellular stores. Phospholipase C (PLC) activation by the TRPM7 kinase domain can increase inositol triphosphate (IP3) formation and the consequent calcium liberation from intracellular stores, which could modify many calcium-mediated responses. Further experiments are required to clarify the precise contribution of each factor and in what conditions and to what extent they can influence cell death induced by retinal ischemia. These experiments should include intracellular calcium measurements and TRPM7 silencing experiments to confirm their role in the neuronal cell death after an ischemic insult.

Regardless of the mechanism of damage, our results showing increased expression of TRPM7 channels suggest their possible contribution to ischemic damage in the retina as part of the non-glutamatergic mechanisms of cell death. Several works from other authors have explored the therapeutic potential of the inhibition or blockade of TRPM7 in an ischemic episode in different models [14,18,19,50,51]. In cultured murine neurons with prolonged glucose deprivation, the suppression of TRPM7 increased their survival [50]. In experiments performed in rat renal I/R, inhaled anesthetic sevoflurane, which regulates the expression of TRPM7, reduced the levels of apoptosis and oxidative stress [19]. Moreover, TRPM7 deletion in GABAergic neurons has demonstrated a protective effect [18]. In this sense, experiments of gene silencing with siRNA also decreased neuronal cell death in cerebral ischemia [14]. In a mouse model of intracerebral hemorrhage, the upregulation of TRPM7 was related to an increase in the calcium overload and increased damage [51].

Before its consideration as a therapeutic target, some issues concerning the TRPM7 channels need to be addressed: its possible interaction with other channels, their precise role in human diseases, and the identification of specific agonists and antagonists acting on the ionic channel or on the kinase domain [31]. To date, several molecules are known to regulate TRPM7 expression (such as nerve growth factor (NGF), interleukin (IL)-18, angiotensin (Ang) II, transforming growth factor (TGF)-β1, D-Glucose and the platelet derived growth factor (PDGF)-BB) by interacting with receptors that activate specific signaling pathways. Moreover, several microRNAs (miR-135a, miR-149 and miR-543) can decrease the expression of TRPM7 [31]. Other molecules such as aldosterone, epidermal growth factor and bradykinin act on the kinase domain and stimulate the signal transduction and phosphorylating functions of TRPM7 [31]. To achieve effectiveness, the use of TRPM7 as a therapeutic agent in ischemic-associated retinal diseases will probably require addressing more than one cell death pathway with the combined use of other drugs, quite probably those acting against glutamatergic death mediators. In this sense, it has been previously shown that inhibiting multiple forms of cell death optimizes ganglion cell survival after retinal ischemia reperfusion injury [52].

In conclusion, our work strongly suggests that retinal ischemia induces Müller cell gliosis and increases TRPM7 channels in Müller cells. TRPM7 overexpression, along with the immunoreactive process involving Müller cell gliosis (which can include the release of proinflammatory cytokines such as TNF-α, an excess production of NO, the disruption of the homeostasis and the triggering of direct or indirect cytotoxic effects [53]), could be promoting retinal dysfunction after the I/R process. This suggests that TRPM7 could arise as a new drug target to be explored in retinal pathologies associated with ischemia.

## 4. Materials and Methods

### 4.1. Animals

Adult male and female C57BL/6J mice with ages ranging from 8 to 10 months of age were employed. Mice were kept under controlled conditions of temperature (23 ± 1 °C), humidity (55–60%), and light in a cycle of 12 h light/12 h darkness. At the end of the experiment, all animals were euthanized by cervical dislocation. All animals were handled and maintained following the current European Community Council Directive 86/609/EEC as well as ARVO Statement for the Use of Animals in Ophthalmic and Vision Research and European Directive 2010/63/EU. All procedures and protocols for this study were approved and reviewed by the Ministry of Agriculture, Environment, Climate Change, and Rural Development from Autonomic Government of Valencia. The assigned code is 2019/VSC/PEA/0266.

### 4.2. Ischemia-Reperfusion

Mice were divided into two groups according to the experimental procedure: the ischemia/reperfusion group (I/R) (*n* = 5) and age-matched mice for the control group (*n* = 5). This sample size was calculated for a 90% probability to obtain statistically significant differences with a *p* value < 0.05. For the I/R group, mild acute transient retinal ischemia was induced by increasing the intraocular pressure (IOP) in the anterior chamber for 30 min on the left eyes. For that purpose, mice were anesthetized with isoflurane and the anterior chamber was cannulated with a 33-gauge needle connected to a normal saline container placed 1.8 m above the animal level. After the IOP was increased for the scheduled time, the needle was removed to allow the retinas to be reperfused. In the ischemic group, ischemia was induced in the left eyes while the right eyes were used as internal controls. After 72 h of reperfusion, animals were euthanized and the eyes were enucleated and processed.

### 4.3. Electroretinograph Recordings

Electroretinography (ERG) responses were recorded under complete darkness following overnight adaptation. The animals were anesthetized with ketamine (100 mg/kg, i.p.) and xylazine (4 mg/kg, i.p.) and placed on a thermal blanket set at 38 °C for bilateral recording. Tropicamide (1%, Alcon Cusí, Barcelona, Spain) was used to dilate the pupils, and polyacrylic acid carbomer (0.2%, Viscotears, Novartis, Barcelona, Spain) was applied to enhance electric contact and prevent eye dehydration. DTL fiber electrodes (Retina Technologies, Scranton, PA, USA) were employed for recording, while a 25 g platinum needle inserted under the scalp between the eyes served as a reference electrode. A ground needle electrode was placed under the skin in the base of the tail. The entire procedure took place within a Faraday cage. Scotopic ERG responses were recorded in both eyes using a Ganzfeld illuminator that generated flashlight stimuli. A total of eleven stimuli with progressively increasing luminance and a duration of 10 ms were presented to the animals. A 10 s interval separated the dimmer flashes (−5 to −0.6 log cd·s/m^2^), while a 20 s interval was provided between the brightest flashes (0–1 log cd·s/m^2^). Signal amplification and band-pass filtering (1–1000 Hz, without notch filtering) were performed using a DAM50 data acquisition board (World Precision Instruments, Aston, UK). Stimulus presentation and data acquisition (4 kHz) were carried out using a PowerLab system (AD Instruments, Oxfordshire, UK). The amplitude of the a-wave, measured from the baseline before the stimulus to the most negative trough, and the amplitude of the b-wave, measured from the trough of the a-wave to the peak of the b-wave, were quantified.

### 4.4. Optical Coherence Tomography

The retinal structure and the vascular plexuses were analyzed in vivo using SPECTRALIS OCT and the angiography module (version 6.9.4.0, Heidelberg Engineering Inc., Franklin, TN, USA). Intraperitoneal injections of ketamine (100 mg/kg) and xylazine (4 mg/kg) were used to anesthetize the animals and 1% tropicamide (Alcon Cusí) was topically administered to cause mydriasis. To improve the quality of the images and preserve the ocular surface during the test, a contact lens and physiological serum were placed on the cornea. High-resolution OCT images and OCTA dense scans were obtained in the central retina above the optic nerve before the procedure and 72 h after ischemia in both groups. The thicknesses of the total retina, the complex retinal nerve fiber layer (RNFL)–ganglion cell layer (GCL)–inner plexiform layer (IPL), the inner nuclear layer (INL) and the outer plexiform layer (OPL) with the outer nuclear layer (ONL) were measured in the optic nerve in ten different points the complex retinal nerve fiber layer (RNFL)–ganglion cell layer (GCL)–inner plexiform layer (IPL), each separated by 500 μm using ImageJ (version 1.52n, National Institutes of Health, Bethesda, MD, USA). The vascular density, the junction density and the lacunarity of the superficial vascular plexus were evaluated with AngioTool software (0.6a version, National Cancer Institute, Bethesda, MD, USA) [54]. The junction density gives information about the ramifications of the vessels, whereas the lacunarity measures the sizes of gaps surrounding the vessels.

### 4.5. Immunohistochemistry

All histological studies were performed 72 h after ischemia. After euthanasia of the animals, the dorsal margin of the limbus was marked by a suture. The eyes were enucleated and fixed at room temperature in 4% paraformaldehyde (PFA) for 1 h, washed in 0.1 M phosphate buffer (PB; pH 7.4) and sequentially cryoprotected in 15%, 20% and 30% (*w*/*v*) sucrose. The cornea, lens and vitreous body were removed, and the eyecups were prepared for cross-sectional cryosections as previously described by our group [55,56]. Sections of 16 μm thickness were obtained and stored at −20 °C.

For single or double immunostaining procedures, sections were thawed, washed in PB and incubated for 1 h in blocking solution consisting of 10% (*v*/*v*) normal donkey serum in PB with 0.5% Triton X-100 (Sigma-Aldrich, St. Louis, MO, USA). The sections were then incubated overnight at room temperature with the appropriate combinations of primary antibodies at different dilutions in PB with 0.5% Triton X-100. Sections were then washed in PB and incubated with secondary antibodies for 1 h. Finally, sections were washed in PB and mounted with Citifluor (Citifluor Ltd., London, UK) under a coverslip. Images were taken with a Leica TCS SP8 confocal laser-scanning microscope (Leica Microsystems, Wetzlar, Germany).

### 4.6. Antibodies

The primary antibodies used were Rabbit anti-TRPM7 (1:100, Alomone, Jerusalem, Irsael ref.: ACC-047); mouse anti-CRALBP (1:100, Abcam, Cambridge, UKref.: ab15051); guinea pig anti-GFAP (1:200, Synaptic Systems, Göttingen, Germany ref.: 173 004). The secondary antibodies used in this work were Donkey anti-rabbit conjugated to Alexa-488 (ref.: A21206); donkey anti-mouse conjugated to Alexa-555 (ref.: A31570); donkey anti-guinea pig conjugated to Alexa-633 (ref.: A21105). All were from ThermoFisher Scientific, Whaltham, MA, USA and used at 1:100.

### 4.7. Data Quantification

To obtain data on the amount of TRPM7 fluorescence, confocal microscopy images were captured from two non-consecutive whole sections of each individual without modifying the laser intensity or gain parameters. Subsequently, square regions of 250 μm × 250 μm were analyzed at intervals of 500 μm from the center of the retina towards the periphery (4 nasal and 4 temporal). Fluorescence quantification was performed using ImageJ software, where the images were binarized, the “plot profile” function was utilized and the area under the curve corresponding to Müller cells was measured. Finally, each of the results was normalized to the mean of the control samples.

### 4.8. Statistical Analysis

Statistical analysis was performed using GraphPad Prism (version 6.0.0, GraphPad Software, San Diego, CA, USA). Kolmogorov–Smirnov or Shapiro–Wilk tests were used to assess the normality, and the non-parametric U Mann–Whitney test was carried out to analyze the differences among groups. The effects of ischemia-reperfusion were evaluated with a two-way ANOVA test. A *p* value of less than 0.05 was considered significant.

## Data Availability

Data and images generated during the current study are available from the corresponding author on reasonable request.
